# Exploring reasons and motivations for suicide attempts in prison before and during the SARS-CoV-2 pandemic

**DOI:** 10.1186/s40352-025-00380-2

**Published:** 2025-11-11

**Authors:** Leonel da Cunha Gonçalves, Laurent Gétaz, Patrick Heller, Kelly Gonçalves, Judith Sultan, Diane Golay, Anja J. E. Dirkzwager, Hans Wolff, Stéphanie Baggio

**Affiliations:** 1 Division of Prison Health, Geneva University Hospitals, Geneva, Switzerland; 2 Institute of Primary Health Care (BIHAM), University of Bern, Bern, Switzerland; 3 Division of Tropical and Humanitarian Medicine, Geneva University Hospitals, Geneva, Switzerland; 4 University of Geneva, Geneva, Switzerland; 5 Faculty of Law and Criminology, Vrije University Amsterdam, Amsterdam, Netherlands; 6 Institute of Psychology, University of Lausanne, Lausanne, Switzerland

**Keywords:** Suicide attempts, Prison, Mental and physical health, COVID-19

## Abstract

**Background:**

Suicide attempts represent a critical public health concern in prison settings, where rates are substantially higher than in the general population. The COVID-19 pandemic introduced additional stressors, yet little is known about its impact on suicide attempts among detained persons. This study aimed to identify the underlying reasons and motivations for suicide attempts in a Swiss pre-trial prison and to examine changes before and during the pandemic. We analyzed 205 suicide attempts by 125 detained persons between 2016 and 2021. Data were collected from clinical and prison records. Reasons and motivations were extracted using content analysis. Population-averaged logistic regression models were used to examine differences between periods.

**Results:**

Suicide attempts were associated with health-related and personal issues (85%), prison-related problems (76%), and interpersonal conflicts (61%). Psychological distress, juridical issues, and conflicts with correctional officers were the most common reasons. Motivations included protest against the institution (39%), desire to die (18%), escape (11%), and help-seeking (7%). There was an increase in health-related and personal problems during the pandemic, particularly dissatisfaction with medical care (+104%), physical pain (+181%), and psychological distress (+18%), while help-seeking motivations decreased (-72%). Psychiatric morbidity and self-harm history were associated with these outcomes. Sample characteristics remained largely stable across periods.

**Conclusions:**

This study highlights the multifaceted nature of suicide attempts in prison and the impact of the COVID-19 pandemic on health-related and personal issues. While preventive measures were essential for infection control, they may have increased psychological distress, and reduced medical resources likely exacerbated clinical needs. These findings underscore the importance of balancing public health measures with continuous access to care during public health emergencies.

## Introduction

Suicide attempts pose a considerable public health concern, particularly among persons in detention (Favril et al., 2022). Suicide is a leading cause of mortality in prisons in high-income countries and has profound consequences on the mental health and well-being of incarcerated individuals, as well as their families and relatives (Blaauw et al., 2001; Gauthier et al., 2015). Additionally, it impacts the welfare of correctional staff, institutional functioning, and the overall perception of the correctional system (Way et al., 2005). The coronavirus (COVID-19) pandemic has further exacerbated the challenges faced by correctional systems worldwide and it is known that infectious disease outbreaks can lead to an increase in suicides (Mukhtar & Candilis, 2022; Zortea et al., 2021). Yet the specific impact of the COVID-19 on prison suicide and suicide attempts remains understudied (Radeloff et al., 2021). Understanding the reasons and motivations behind suicide attempts in prison—before and during the SARS-CoV-2 pandemic—could help in developing prevention strategies to protect those living and working in detention facilities during times of crisis. This need is even more urgent given that the annual likelihood of extreme epidemics is expected to triple in the coming decades (Marani et al., 2021).

### Suicide and suicide attempt before and during COVID − 19

Suicide is often one of the leading causes of death in prison and occurs at rates significantly higher than in the general population. A meta-analysis of data from 82 jurisdictions worldwide, spanning the period from 2000 to 2021, found that most countries reported prison suicide rates ranging between 24 and 89 per 100,000 person-years, with substantial cross-national variation (Mundt et al., 2024). Rates were markedly higher in Europe, in high-income countries, and in nations with lower incarceration rates. Another meta-analysis, based on data from 36 countries, evidenced that the prevalence of suicidal behavior in low- and middle-income countries (LMIC) was lower than in high-income countries, while the prevalence of self-harm acts was higher (Aon et al., 2025). In several high-income countries, suicide accounts for nearly half of all prison deaths (Berman & Canning, 2022; Bukten & Stavseth, 2021; Carson & Cowhig, 2020; Gentile et al., 2021), whereas in LMIC it represents only about 10% (Aon et al., 2025).

Within Europe, Switzerland has particularly high prison suicide rates. According to the Council of Europe Annual Penal Statistics – Prisons and Penal Institutions I survey (Aebi & Cocco, 2024), the average suicide rate across European prisons in 2022 was 71 per 100,000 detained persons. In contrast, Switzerland recorded a rate of 202 per 100,000, the second highest rate on the continent. This figure is approximately 20 times higher than the suicide rate in the general Swiss population (Federal Statistical Office., 2022). In 2022, suicides accounted for 76% of all deaths in Swiss prisons (Federal Statistical Office., 2024).

Despite growing concern, studies examining suicidal behaviors and attitudes in prison before and during the COVID-19 pandemic remain scarce. In Switzerland, a study conducted in the country’s largest pre-trial prison reported a 57% increase in the relative risk of suicide attempts during the pandemic compared to the pre-pandemic period (Gétaz et al., 2021). Similarly, research from Italian prisons found that individuals incarcerated after the onset of the pandemic exhibited higher levels of suicidal intent than those admitted before (Santoriello et al., 2024). Increases in prison suicides were also reported in certain U.S. jurisdictions, such as California (California Department of Corrections and Rehabilitation., 2023). In contrast, prisons in the United Kingdom saw a 25% relative reduction in self-harm rates following the implementation of the lockdown measures (Hewson et al., 2021).

In comparison to the limited data available in prison settings, research among the general population is more extensive. Meta-analyses suggest that although suicide mortality remained stable—or even declined slightly—during the initial phase of the pandemic (Pirkis et al., 2021; Yan et al., 2023), rates of self-harm, suicidal ideation, and suicide attempts increased when compared to pre-pandemic levels (Dubé et al., 2021; Yan et al., 2023).

### Correlates, reasons, and motivations for suicide attempts

Previous studies—primarily based on quantitative data—have identified a broad range of risk factors associated with suicide attempts among incarcerated individuals. A meta-analysis of 20 studies from as many high-income countries (Favril et al., 2022) reported socio-demographic (unemployment, low educational attainment, and being single), criminological (violent offenses, longer sentences, previous incarceration), clinical (suicidal ideation, histories of self-harm and suicide attempts, psychiatric conditions, use of psychotropic medication, impulsivity, and substance abuse), and historical adversities (childhood trauma, out-of-home care, and family histories of suicide or self-harm) as risk factors. In LMIC, the strongest associations with suicide attempt were female sex, recidivism, and family history of mental illness (Aon et al., 2025).

Environmental factors within prison may further amplify vulnerability. Contextual stressors such as solitary confinement, peer victimization, lack of social support, disciplinary sanctions, and institutional unemployment have been linked to suicide attempts (Favril et al., 2022). Additional studies from Switzerland have underscored the role of overcrowding and high turnover as contributor factors (Baggio et al., 2018; Wolff et al., 2016).

While the body of research on quantitative correlates is extensive, considerably less attention has been given to the subjective experiences and underlying motivations of persons who attempt suicide while incarcerated, which limits our understanding of the personal and contextual meanings behind such acts. Existing qualitative studies provide valuable, yet still scarce, insights. For example, a study in United States identified mental health challenges (e.g., depression, hallucinations, impulsivity), interpersonal tensions, and prison-specific stressors as key contributors to suicide attempts (Suto & Arnaut, 2010). In England and Wales, other studies similarly found that suicide attempts were often linked to traumatic life events, procedural uncertainties (e.g., upcoming trials or parole decisions), mental health struggles, and situational frustrations (e.g., lack of tobacco or placement in segregation) (Marzano et al., 2011; Rivlin et al., 2013).

The motivations behind suicide attempts in prison are diverse and often ambivalent. One study reported that while most individuals intended to die (73%), many described their actions as impulsive (40%) (Rivlin et al., 2013). Another study highlighted a desire for peace as a common theme (Marzano et al., 2011). In an Australian study, 43% of suicide attempts were aimed at achieving psychological relief, 32% sought to escape intolerable circumstances, and 24% served instrumental purposes, such as eliciting attention or influencing one’s situation (Dear et al., 2000). Control-seeking motivations were similarly noted in another U.S. study, suggesting that suicide attempts can also represent a means of exerting agency in an otherwise highly controlled environment (Suto & Arnaut, 2010).

### The present study

Despite extensive research quantifying suicide rates and associated risk factors in prison settings, the subjective reasons and motivations behind these acts remain underexplored (Suto & Arnaut, 2010). Moreover, most research focuses on death by suicide, providing limited insight into the psychological and situational factors experienced by those who survive attempts (Rivlin et al., 2013). In addition, existing research on reasons and motivations for prison suicide attempts is relatively dated, largely derived from Anglo-Saxon countries, and often involves small and gender-specific samples, which limits the generalizability of findings to other prison systems. This gap is a crucial limitation, as understanding the lived experiences and personal meanings attributed to suicide attempts in prison is essential for designing effective prevention strategies.

The relevance of this research is also underscored by the notably high suicide rates in Swiss prisons and the observed increase in suicide attempts in some correctional settings during the COVID-19 pandemic. Yet, the specific explanations for these trends remain unclear, especially from the perspective of the individuals directly affected. By collecting data from the institutional records of 125 men and woman detained in Switzerland and employing a mixed-methods content analysis, in which qualitative themes were first identified from clinical records and then quantified through coding for subsequent statistical analysis, the aims of this study were: (a) to identify the reasons and motivations behind suicide attempts in prison, and (b) to examine differences in these outcomes before and during the pandemic. The findings of this study are expected to deepen the understanding of the suicidal process within prisons, highlight potentially modifiable triggers, and ultimately inform tailored, culturally relevant suicide prevention strategies that can improve the well-being of people living and working in detention facilities.

## Methods

### Context

In Switzerland, the organization of health services varies from canton to canton (Chatterjee et al., 2019). In Geneva, health care services are independent of the prison authorities, being provided by the cantonal university hospital. Mental health care is provided in case of court-ordered psychiatric treatment, at the request of detained persons, assessed upon prison entry (e.g., in case of suicidal ideation or substance use problems), mandated by medical staff, when detained persons are serving isolations measures, or in case of emergencies. Detained persons who attempt suicide are assessed by the on-call psychiatrist or transferred in emergency to the cantonal hospital, who also possesses an inpatient prison unit. However, as in other countries, resources for prison mental health care are limited. This means that detained persons with mental health issues who do not request care and do not disrupt the prison functioning are unlikely to be treated.

The COVID-19 pandemic has disrupted mental health services to an unprecedented degree, while the psychological distress and demand for mental health support have increased (Duden et al., 2022). During the pandemic, assessment of suicide risk, referral for suicidal ideation, and hospitalization after suicide attempts decreased (Hewson et al., 2020). Additionally, inpatient treatment capacities and the length of hospital stays were often reduced (Fasshauer et al., 2021; Fasshauer, Bollmann, Hohenstein, Hindricks, Fasshauer et al., 2021a, b). However, mental health services have regained capacity after the initial outbreaks (Villarreal-Zegarra et al., 2023).

The data were collected from the pre-trial prison of Geneva, Switzerland. It is the largest remand custody facility in the country, with 398 detention places. However, the prison is constantly overcrowded (average occupancy rate of 175% between 2013 and 2019) (Gétaz et al., 2022) and has a high turnover rate (on average 73% between 2013 and 2017, calculated as the number of releases divided by the number of admissions plus the average prison population of the previous year) (Baggio et al., 2018). By January 2020, there was an average of 526 persons detained in this facility. Most detained persons are foreigners and spend 23 h a day in their cell. Despite being initially designed for the detention of men in remand custody, there are also sentenced individuals and women (with 40 allocated places) in the institution. A prior study conducted in the current research site evidenced that, during the pandemic period, prison occupancy rate decreased (141% occupancy in 2020), yet the relative risk of suicide attempts rose significantly, from an average of 4.4% between 2016 and 2019 to 7.0% in 2020 (Gétaz et al., 2021). While this finding may not be generalizable to later periods or broader contexts due to evolving COVID-19 measures and changing prison conditions, it underscored the clinical relevance of the present study.

### Procedure

Data were collected between June 2021 and October 2022 from prison and clinical records. A list of all acts of self-harm between January 2016 and July 2021 (*n* = 243) was recorded by the prison nurses, which was used to identify suicide attempts. In this study, a “suicide attempt” was defined as a self-initiated act of self-harm with a high potential to result in death—including hanging/strangulation and massive abuse of medication—regardless of confirmed suicidal intent. The determination of “high potential to result in death” was made by one of the study authors (PH), who is the head of the prison psychiatric services in Geneva, based on clinical judgment. This lethality-based operationalization aligns with the prison’s clinical protocols and documentation practices. While this diverges from the DSM-5-TR (American Psychiatric Association, 2022) definition, which emphasizes intent, our approach prioritizes the clinical and forensic significance of acts that posed a serious risk to life. Self-harm behavior that did not cause a serious risk to life (*n* = 35, e.g., cutting, foreign body ingestion, non-massive ingestion of medication, and hanging threats and simulations) and acts that resulted in death (*n* = 3) were excluded, resulting in a final sample of 205 suicide attempts.

All the data collected for this study were extracted from clinical records and were checked by at least two study authors for accuracy. Extracted data were recorded in REDCap^®^ (Harris et al., 2009) and then exported to the software Stata 17 (StataCorp., 2021). Suicide attempts (the unit of analysis in this study) were divided into two groups: incidents that occurred before or during the COVID-19 period. For the purposes of this study, COVID-19 corresponds to the period from March 16th, 2020 (date at which the lockdown was implemented in Switzerland), to July 31 st, 2021 (corresponding to the end of the third COVID-19 wave; Roelens et al., 2021).

The reasons and motivations behind the suicide attempts were mostly derived from the emergency psychiatric reports generated by the local hospital (independent from the prison institution), generally within 24 h following the events. These psychiatric assessments involve direct interaction with the patient as well as a review of available clinical data. Patients are referred to the hospital in cases of severe suicide attempts that require medical monitoring, either for psychiatric or somatic care. Referrals may also occur at night when no psychiatrist is available in the prison. Persons who are not transferred to the hospital are assessed on site by the prison medical staff through direct contact with the patient. Data were also collected from other clinical records, based on the reports of healthcare professionals (e.g., psychologists, nurses and medical doctors), for the four weeks preceding and following the suicide attempts. In cases where no records were available within this window, information from slightly earlier or later periods was consulted. In Geneva, all medical records—including both those from prison healthcare services and from hospitals outside prison—are integrated into a unified electronic health record system (*Dossier Patient Informatisé*, DPI), which was fully available to the authors.

The categorization of reasons and motivations was carried out by the study authors during data extraction, based on the information available in DPI. Although self-harm events are systematically recorded by clinical services, information on their reasons and motivations was not standardized since there is no specific questionnaire to assess these events. Therefore, in some cases, information is more complete than others, but it is not exhaustive. Socio-demographic and clinical characteristics were also collected from DPI. Data for the criminological variables was provided by the prison authorities.

### Variables

The reasons for the suicide attempts answered *because of what* the suicide attempts were committed, while the motivations for the suicide attempts refer to *for which purpose* the acts were made. That is, reasons refer to contextual or precipitating factors leading to the suicide attempt, while motivations describe the intended purpose or goal of the act. Reasons that appeared less than five times across the clinical records (e.g., financial problems) were not included in the findings. These outcome variables (see results section) were binary, with a value of 1 indicating the presence of a particular theme, and 0 otherwise.

Socio-demographic, criminological, and clinical variables were also collected. Socio-demographic variables consisted of age (in years), sex (0 = female, 1 = male), and region of origin (0 = non-Middle East and North Africa [MENA], 1 = MENA; because MENA countries represented the largest group in our sample). Criminological variables comprised detention status (0 = sentenced, 1 = pre-trial), having committed a violent crime (crimes involving the use of force or threat of force against others, 0 = no, 1 = yes), type of cell allocation (0 = individual cell, 1 = shared cell), and receiving prison visits (0 = no, 1 = yes). Clinical variables included self-harm history (before and during detention, including previous suicide attempts, 0 = no, 1 = yes), the count of different mental health problems,^1^ the count of mental health consultations before the suicide attempt, the count of different psychotropic medications prescribed in prison,^2^ and time from entry in prison until the suicide attempt (in days). In addition, a variable indicating the COVID-19 period (i.e., suicide attempts committed before [= 0] or during COVID-19 pandemic [= 1]) was generated.

### Analyses

Content analysis was used to identify the reasons and motivations behind suicide attempts in prison (objective 1). The procedure was based on that proposed by Krippendorff (2019): (1) familiarization with the data, (2) developing a coding scheme to classify the content, (3) applying the coding scheme to the data, and (4) analyzing the results and identifying themes within the data. After reading all information regarding the suicide attempts, two study authors (LCG and SB) developed a coding scheme and independently coded the data in the defined categories. The agreement between coders, calculated using Krippendorff’s alpha (1970) reliability estimate (α) across all coding categories was good (α = 0.86 [0.77, 0.94], values above 0.80 are almost perfect). Any coding disagreement was resolved through discussion between authors.

We described the characteristics of the sample at the person level and at the suicide attempt level using percentages and frequencies for dichotomous variables, and median with interquartile ranges for continuous and count variables. Differences in these characteristics between periods at the time of the first suicide attempt were assessed by regressing each variable on the dichotomous COVID-19 predictor using bivariable regression models (linear, negative binomial, or logistic depending on the outcome type).

To examine differences in reasons and motivations for suicide attempts before and during the COVID-19 pandemic (objective 2), each outcome was regressed on the dichotomous COVID-19 predictor using population-averaged logistic regression models for panel data estimated with generalized estimating equations (GEE) (Liang & Zeger, 1986). An exchangeable working correlation structure with robust standard errors was specified. This approach accounts for clustering of multiple attempts within individuals. GEE models were chosen because they provide population-level estimates, which are particularly relevant for public health interpretation as they reflect average effects across the sample rather than person-specific effects (Hubbard et al., 2010). They also yield more stable estimates for categories with small cell counts (Hardin & Hilbe, 2012). Reasons and motivations with fewer than 10 cases were not included in inferential analyses to preserve statistical power. The relative increase in the outcomes between periods was also calculated.

Bivariable regressions were conducted to indicate the direction and strength of associations, followed by multivariable models to reduce the risk of confounding. Selected covariates for the multivariable models included those identified in the meta-analysis of Favril et al. (2022) that were available in our dataset (i.e., violent crime, visits, self-harm history, and mental health problems). Age and region were added to account for socio-demographic differences. Finally, we included the number of days from prison entry until suicide attempt to control for time of exposure to the outcome.

All analyses were performed in Stata 17 (StataCorp). Statistical significance was set at α = 0.05. Missing data on study covariates (ranging from 0.0% to 9.2%) were imputed manually using predicted probabilities from regression models fitted separately for each missing item within each detained person.^3^ No multicollinearity was observed among variables selected for multivariable analyses (all variance inflation factors < 1.2).

## Results

### Descriptive characteristics of the sample

The study sample included 125 individuals, corresponding to 205 suicide attempts, of which 129 (62.9%) were committed before the COVID-19 pandemic and 76 (37.1%) during the pandemic. Each person committed between 1 and 9 suicide attempts (Median = 1, inter-quartile range [IQR] = 1) and 6 persons (4.8%) had attempts in both periods. There was a higher proportion of suicide attempts involving strangulation (62.4%) compared to those involving the abuse of medication (37.6%).

When considering the first suicide attempt (See Table 1 – Person level), the age of the participants ranged from 18 to 72 years (Median = 29, IQR = 12). The majority were men (90.4%) and from MENA countries (60.8%), with only 8 (6.4%) being Swiss citizens.^4^ Most persons were in pre-trial detention (62.4%), accused of non-violent crimes (74.4%), allocated to a shared prison cell (76.8%), and had not received visits (Median = 0, IQR = 0) during the median of 221 days of stay the institution.

**Table 1:** Descriptive characteristics of the sample

Variables	Person level^a^ *N* = 125		Suicide attempt level^b^ *N* = 205	
	Median/%	IQR/*n*	Median/%	IQR/*n*
*Socio-demographic*				
Age	29	12	31	10
Men	90.4	113	93.7	190
MENA	60.8	76	62.9	129
*Criminological*				
Pre-trial	62.4	78	58.5	120
Violent crime	25.6	32	33.7	69
Shared cell	76.8	96	75.6	155
Visits	0	0	0	0
Duration of stay (days)	221	202	239	221
*Clinical*				
Self-harm history	75.2	94	84.4	173
No. mental health problems	3	1	4	2
No. psychotropic medications	3	2	3	2
No. mental health consultations	0	4	1	7
Days until suicide attempt	89	136	98	129

*SD S*tandard deviation, *n * partial sample size, *IQR *Interquartile range, *MENA *Middle East–North Africa

^a^Person level characteristics correspond to the first suicide attempt

^b^Suicide attempt level variables are the variables used for inferential analyses

Clinically, 75.2% had a history of self-harm and 99.2% had some type of mental health problem, with a median of 3 different symptoms (IQR = 1, range 0–7) and 3 different psychotropic medication categories (IQR = 2, range, range 0–5) prescribed. Most had received no received mental health consultations in prison prior to their suicide attempt (Median = 0, IQR = 4, range 0–78). Although the first suicide attempt occurred at a median of about three months after prison entry (Median = 89 days, IQR = 136, range 1–678), 25% of the events occurred during the 30 days of entry in prison.

There were no significant differences in the characteristics of the sample before and during the pandemic, except for age. During the COVID-19 period, the sample was slightly younger, with a median age of 28 years (IQR = 14), compared to 31 years (IQR = 10) before the pandemic (*p* = 0.003).

### Reasons and motivations for suicide attempts

The reasons and motivations for suicide attempts, as well as their percentages and frequencies, are presented in Table 2. A total of 13 reasons were identified and grouped into three different themes: (1) health-related and personal issues (84.9%), (2) prison-related problems (75.6%), and (3) interpersonal relations (60.5%). *Health-related and personal issues* included psychological distress (72.7%), sadness and hopelessness (31.7%), dissatisfaction with medical care (21.5%), physical pain (7.8%), and psychosis (4.4%). *Prison-related problems* included juridical issues (34.6%), detention conditions (27.8%), solitary confinement (26.8%), and penal issues (12.7%). *Interpersonal relations* included correctional officers (40.0%), family and relatives (24.9%), co-detained persons (13.2%), and other staff (8.3%). COVID-19 (2.4%) was not included in these themes because it was related to the three of them. Only one of the 205 events did not have a discernible reason. Each suicide attempt had a median of 3 (IQR = 2, range 0–9) underlying reasons.

**Table 2:** Reasons and motivations for suicide attempts

Variables	%	*n*
*Reasons*		
Health-related and personal issues	84.9	174
Psychological distress	72.7	149
Sadness and hopelessness	31.7	65
Dissatisfaction with medical care	21.5	44
Physical pain	7.8	16
Psychosis	4.4	9
Prison related problems	75.6	155
Juridical issues	34.6	71
Detention conditions	27.8	57
Solitary confinement	26.8	55
Penal issues	12.7	26
Interpersonal relations	60.5	124
Correctional officers	40.0	82
Family and relatives	24.9	51
Co-detained persons	13.2	27
Other staff	8.3	17
Other		
COVID-19	2.4	5
*Motivations*		
Protest	38.5	79
Desire to die	17.6	36
Escape	11.2	23
Seeking help	6.8	14

*n *partial sample size, *N = *205

Four motivations for suicide attempts were identified. Most events were made as a protest against the prison institution (38.5%). Other motivations included a desire to die (17.6%), escape (11.2%), and seeking help (6.8%). There was no discernible motivation in 30.7% of the events and 4.9% had a dual purpose (Median = 1, IQR = 1, range 0–2). A definition of each reason and motivation for suicide attempt is presented in Table 3.

**Table 3:** Definition of the reasons and motivations for suicide attempts

Reasons and motivations for suicide attempts	Definition
*Reasons*	
Health-related and personal issues	
Psychological distress	Intense emotional suffering that may manifest as internalized states (such as anxiety, frustration, or ruminative worry) or externalized responses (such as impulsive acts or reactive outbursts). Often triggered by an inability to cope with adverse life events, interpersonal conflict, or demanding environments.
Sadness and hopelessness	Includes a range of depressive emotions such as loneliness, desperation, exhaustion, and feelings of worthlessness or abandonment. Often associated with a chronic sense of suffering or personal breakdown.
Dissatisfaction with medical care	Refers to negative perceptions or experiences related to health care access or quality, including unfulfilled requests for assessment or treatment, refusal of medication, or perceived indifference from medical staff.
Physical pain	Involves the experience of bodily discomfort or suffering, reported in various areas such as the head, chest, abdomen, back, or limbs. Physical pain may act as a trigger or contributing factor to psychological distress.
Psychosis	Refers to the presence of hallucinations, including auditory or visual, which significantly impair reality testing.
Prison related problems	
Juridical issues	Legal and procedural concerns related to sentencing, trials, detention status (e.g., remand, expulsion), parole, or access to legal representation. Includes feelings of injustice, confusion, or helplessness about one’s legal process.
Detention conditions	Dissatisfaction with general living conditions in prison, such as overcrowding, lack of hygiene, insufficient privacy, reduced autonomy, limited social contact, restrictions on activities, or feelings of insecurity.
Solitary confinement	Psychological and emotional impact of being placed in isolation as punishment for rule violations. Involves coping difficulties related to social and sensory deprivation.
Penal issues	Concerns about how the prison sentence is administered, including cell or unit transfers, treatment by staff, or a perceived lack of fairness within the institution itself.
Interpersonal relations	
Correctional officers	Conflicts with correctional officers, including victimization (e.g., violence, sexual assault, insults, mockery), perceived maltreatment, intrusive behavior, lack of support or responsiveness to personal needs, and perceived unfair disciplinary actions.
Family and relatives	Emotional distress related to family or intimate relationships, including concerns for loved ones’ well-being, separation due to incarceration, lack of contact or support, relationship breakdowns, and conflicts.
Co-detained persons	Conflicts or victimization involving other incarcerated individuals, such as physical or sexual violence, extortion, threats, verbal abuse, mockery, and interpersonal disputes.
Other staff	Dissatisfaction with interactions with non-custodial prison staff (e.g., medical, mental health, social workers), including perceived neglect, lack of empathy or support, or unmet expectations in care or assistance.
*Motivations*	
Protest	Suicide attempts used to draw attention, exert pressure, or express opposition toward the justice system or prison administration. Includes protests against decisions, efforts to gain personal benefits (e.g., transfers, hospitalization, avoiding solitary confinement), or to accelerate the resolution of specific demands.
Desire to die	Suicide attempts motivated by a genuine desire to end one’s life, often linked to feelings of hopelessness, guilt, shame, despair, emotional exhaustion, or a perceived lack of meaning and prospects. May be accompanied by farewell gestures or requests for euthanasia.
Escape	Suicide attempts used as a coping strategy to relieve psychological or physical pain. May aim to reduce internal tension, escape from distressing thoughts or emotions, induce sleep, or achieve a calming, sedative effect.
Seeking help	Suicide attempts intended to signal distress and prompt intervention from prison staff. Often related to unmet mental health needs, legal concerns, or challenges of prison life.

### Reasons and motivations for suicide attempts before and during the COVID-19

Differences in reasons and motivations for suicide attempts are presented in Table 4. Bivariable analyses revealed no significant differences for prison-related problems or interpersonal relations between the two periods. However, suicide attempts linked to health-related and personal issues showed a marginal increase (+ 11.5%, *p* = 0.072). Within this category, significant increases were found for dissatisfaction with medical care (+ 103.9%, *p* = 0.012) and physical pain (+ 180.9%, *p* = 0.038), and a marginal increase for psychological distress (+ 17.7%, *p* = 0.056). With respect to motivations, seeking help showed a marginal decrease during COVID-19 (−72.0%, *p* = 0.086).

**Table 4:** Differences in reasons and motivations for suicide attempts before and during COVID-19

**Variables**	Before (*n* = 129)		During (*n* = 76)		***Change***	Differences		
	**%**	*n*	***%***	*n*		*p*	*p*_adj_	
*Reasons*								
Health-related and personal issues	81.4	105	90.8	69	+ 11.5%	0.072	0.090	
Psychological distress	68.2	88	80.3	61	+ 17.7%	0.056	0.085	
Sadness and hopelessness	31.0	40	32.9	25	+ 6.1%	0.773	0.493	
Dissatisfaction with medical care	15.5	20	31.6	24	+ 103.9%	0.012	0.014	
Physical pain	4.7	6	13.2	10	+ 180.9%	0.038	0.022	1
Prison related problems	78.3	101	71.1	54	−9.2%	0.249	0.329	
Juridical issues	35.7	46	32.9	25	−7.8%	0.773	0.918	
Detention conditions	29.5	38	25.0	19	−15.3%	0.895	0.564	
Solitary confinement	20.9	27	36.8	28	+ 76.6%	0.139	0.144	
Penal issues	15.5	20	7.9	6	−40.6%	0.212	0.175	1
Interpersonal relations	61.2	79	59.2	45	−3.3%	0.725	0.664	
Correctional officers	40.3	52	39.5	30	−2.0%	0.744	0.759	
Family and relatives	20.9	27	31.6	24	+ 51.2%	0.101	0.153	
Co-detained persons	15.5	20	9.2	7	−40.6%	0.628	0.654	1
Other staff	7.8	10	9.2	7	+ 18.0%	0.950	0.740	1
*Motivations*								
Protest	34.9	45	44.7	34	+ 28.1%	0.235	0.346	
Desire to die	17.8	23	17.1	13	+ 3.9%	0.934	0.674	
Escape	11.6	15	10.5	8	+ 47.4%	0.951	0.979	1
Seeking help	9.3	12	2.6	2	−72.0%	0.092	0.065	1

*p*-values from bivariable (*p*) and multivariable (*p*_adj_) population-averaged panel data logistic regressions, with exchangeable working correlations and robust standard errors

1. For these outcomes the number of events was very small (< 10) in certain periods, resulting in limited statistical power. Results for these categories should be interpreted with caution

These differences remained after adjusting for covariates (*p* = 0.090, 0.085, 0.014, 0.022, and 0.065, respectively). In multivariable analyses, a higher number of psychological problems was significantly associated with health-related and personal issues (*p* = 0.020), including psychological distress (*p* = 0.029). Self-harm history was marginally associated with dissatisfaction with medical care (*p* = 0.052).

## Discussion

There is a lack of research on the reasons and motivations behind suicide attempts in detention settings. Although increases in such events were reported in some prisons during the COVID-19 pandemic, the underlying drivers have remained unclear. This study addresses this gap by identifying the main reasons and motivations for suicide attempts and by comparing patterns before and during the COVID-19 period.

Our findings indicate that detained persons who engaged in severe suicide attempts carried a high burden of mental health problems and comorbidity, while often lacking social support. The sample characteristics were largely stable across periods, with the only notable change being a slight decrease in age. This suggests that the observed increase in suicide attempts during the pandemic found in a prior study conducted at the current research site (Gétaz et al., 2021) cannot be explained by socio-demographic, criminological, or clinical differences alone. Suicide attempts in prison appear to result from a complex interplay of health-related and personal problems, prison-related stressors, and interpersonal dynamics. While prison-related and interpersonal reasons remained relatively, health-related problems became more prominent during the pandemic—particularly dissatisfaction with medical care, physical pain, and psychological distress. Psychiatric morbidity and a history of self-harm were further risk factors. Across the study period, most suicide attempts seemed motivated more by protest against the institution than by a genuine intent to die, whereas help-seeking motivations showed a marginal decline during the pandemic.

The content analysis revealed 13 distinct reasons for suicide attempts, corresponding to health-related and personal issues (84.9%), prison-related problems (75.6%), and interpersonal relations (60.5%). These themes align with findings of previous studies conducted in the United States (Suto & Arnaut, 2010) and in England and Wales (Marzano et al., 2011; Rivlin et al., 2013). Among the specific reasons cited, psychological distress, issues involving correctional officers, and juridical concerns were mentioned by more than one-third of participants. Health-related and personal issues, as well as interpersonal difficulties, are well-established risk factors for suicide in the general population (Christensen et al., 2014; Richardson et al., 2021). These factors may carry heightened relevance in prison, given the overrepresentation of individuals with pre-existing vulnerabilities. Conflicts with other detained-persons or prison staff further compound these interpersonal challenges. Moreover, incarceration itself introduces stressors such as legal uncertainty, harsh living conditions, and isolation. Together, these individual and contextual triggers likely contribute to the elevated rates of suicide and suicide attempts observed in detention.

There were four main motivations behind suicide attempts: protest against the institution, a desire to die, escape, and help-seeking. These categories are also consistent with prior studies conducted in Anglo-Saxon prison systems (Dear et al., 2000; Marzano et al., 2011; Rivlin et al., 2013; Suto & Arnaut, 2010). In the general population, two broad categories of motives for suicide attempts have been described: intrapersonal motivations (e.g., escaping overwhelming internal states) and interpersonal motivations (e.g., communication or influence) (May & Klonsky, 2013). In this study, motivations such as the desire to die and escape correspond to intrapersonal factors, while help-seeking and protest align more with interpersonal ones. Protest emerged as the most frequently reported motivation, which highlights the use of suicide attempts in prison as a means of regaining agency or achieving specific outcomes. Such actions are sometimes perceived by staff as manipulative or insincere, leading to a reluctance to respond supportively (Dear et al., 2000). However, individuals reporting protest-related motives often expresse moderate to high suicidal intent (Dear et al., 2000) and protest-driven suicide attempts may inadvertently result in fatal outcomes (Suto & Arnaut, 2010). Misclassifying these attempts as purely strategic may therefore neglect genuine psychological distress and increasing suicide risk.

There was an increase in suicide attempts related to health-related and personal issues during the pandemic. Dissatisfaction with medical care and physical pain were the reasons that increased most, and the only statistically significant changes observed in our study. This highlights potential shortcomings in prison healthcare services during this period. Reduced access to medical services, delays in treatment, and fewer opportunities for supportive contact likely intensified both physical suffering and dissatisfaction with care (Duden et al., 2022; Fasshauer et al., 2021a; Fasshauer, Bollmann, Hohenstein, Mouratis, Fasshauer et al., 2021a, b; Hewson et al., 2020). Medical staff shortages due to COVID-19 exposure, coupled with increased demand and a system-wide focus on pandemic control, may have further compromised the quality and continuity of non-COVID care (Hewson et al., 2020; Kim et al., 2022). As a result, other health needs may have been deprioritized, leaving detained persons with unmet medical needs. These disruptions may partially explain the observed increase in suicide attempts linked to physical pain, supported by the positive association between dissatisfaction with care and pain (Spearman’s ρ = 0.29, *p* <.001, results not presented). The marginal decline in help-seeking motivations may also reflect eroding trust in medical services. These findings underscore the importance of continuity of care during health crises (Mitchell et al., 2020; Moreno et al., 2020).

There was also a marginal increase in suicide attempts associated with psychological distress. Preventive measures implemented during the COVID-19 period in prisons, together with the broader tension and uncertainty of the pandemic, may have contributed to worsening mental health among detained persons. Restrictions on prison visits and the suspension of activities such as work, education, workshops, entertainment, and sports likely intensified negative emotions and behaviors (Gétaz et al., 2022). These disruptions led to feelings of isolation, anxiety about relatives’ well-being, tensions among detained persons, and reduced opportunities for social support, as reported by study participants. Furthermore, many detained persons may lack effective coping mechanisms to manage these additional stressors (Mitchell et al., 2020). Given the well-established link between mental health problems and suicidal behavior (Favril et al., 2020, 2022; Jenkins et al., 2005; Marzano et al., 2011; Zhong et al., 2021), it is not surprising that detained persons with psychiatric morbidity and a history of self-harm—who represented the vast majority of the sample—were at especially high risk of suicide attempts related to personal and health issues. The extremely high burden of mental health problems in this population underscores the need for integrated psychiatric care and systematic suicide prevention protocols.

### Limitations and implications

As with all research, this study has limitations. The data were collected from a single Swiss prison, which limits the generalizability of the findings to other institutions or countries. In addition, some outcome categories, time periods, and covariates involved a limited number of events, reducing statistical power and the precision of estimates. These results should therefore be interpreted with caution. Furthermore, our lethality-based definition of suicide attempts—prioritizing acts with a high potential to result in death—differs from intent-based definitions such as that of the DSM-5-TR (American Psychiatric Association, 2022). As a result, our findings on reasons and motivations may not be fully comparable with studies that define suicide attempts primarily by suicidal intent.

Furthermore, the study relied solely on institutional records, excluding other valuable data sources such as direct accounts from the participants. Reports produced by medical staff may be subject to bias, especially if based on incomplete understanding of the patient’s mental state. Detained persons may also provide distorted or incomplete accounts, whether intentionally or not (Rivlin et al., 2013). Discrepancies between how detained persons describe their suicide attempts and how medical personnel document them have been previously noted (Suto & Arnaut, 2010), and were also evident in our sample. For example, psychiatric emergency reports sometimes emphasized suicidal intent, whereas the detained person described the act as a protest when speaking to other medical staff. To account for such discrepancies, we coded all distinct reasons and motivations reported across different data sources to capture the multifaceted nature of these acts. Moreover, without comparison groups such as non-suicidal self-harm or deaths by suicide, we were unable to examine how reasons and motivations differ across other distress-related behaviors.

Another limitation is the lack of standardized documentation for suicide reasons and motivations in clinical reports, which may also have introduced potential information bias. Despite the good inter-coder agreement on the categories of reasons and motivations that emerged from the data, some may have been misunderstood, misclassified, or not specified. Consequently, the completeness and comparability of the records cannot be guaranteed. These limitations mean that our findings likely reflect only part of the underlying complexity of suicide attempts in prison. Some motivations may remain undocumented or unknown, which restricts the scope of interpretation. This points to the need for standardized reporting protocols for suicide attempts in prison settings, which would both improve clinical documentation and allow for more comprehensive and comparable results.

Despite its limitations, this study provides important insights for suicide prevention in prison settings. For both clinical and research purposes, institutions should incorporate structured fields for documenting reasons and motivations for suicide attempts into standardized reporting forms. This would allow comparability of results, facilitate more targeted interventions, and foster dialogue between staff and detained persons to address underlying issues (May & Klonsky, 2013). Protest-related motives, like all others, should be taken seriously and explored in depth. Psychiatric care and suicide prevention protocols must be integrated, extending beyond crisis response after suicide attempts.

To strengthen prevention capacity, staff training programs such as QPR (Question, Persuade, Refer) or Mental Health First Aid—shown to improve suicide prevention skills in other contexts—could be adapted for correctional environments (Hadlaczky et al., 2014; Litteken & Sale, 2018). Since suicide attempts in prison frequently occur in the context of significant mental health problems, routine psychiatric screening and treatment is central to suicide prevention strategies. Given that most detained persons in our sample were not Swiss, interventions must be adapted to linguistic, cultural, and social diversity. More broadly, prevention strategies should target both individual vulnerabilities and environmental stressors, recognizing that most suicide attempts involved multiple, interacting triggers (Marzano et al., 2011; Rivlin et al., 2013; Suto & Arnaut, 2010).

Importantly, in the context of pandemics such as COVID-19, prison systems must find a careful balance between implementing necessary infection-control measures and maintaining access to essential healthcare services. Disruptions to psychiatric care, psychosocial support, meaningful activities, or visits can exacerbate existing vulnerabilities and heighten the risk of suicidal behavior. Reinforcing prison healthcare during such crises could involve peer support programs, expand use of telemedicine, and facilitating virtual contact with family and relatives. Together, these measures could help preserve continuity of care, alleviate psychological distress, and mitigate suicide risk (Bagnall et al., 2015; Folk et al., 2019; Hewson et al., 2021b).

Future research should deepen understanding of suicide attempts in prisons by incorporating qualitative approaches, enabling more nuanced insights into reasons and motivations beyond what clinical records can capture. Studies that include diverse comparison groups—such as deaths by suicide or non-suicidal self-harm—would further help clarify the processes underlying different self-harm acts. In addition, research should assess the effectiveness of suicide prevention strategies in correctional settings and explore how these interventions can be adapted in culturally relevant ways.

## Conclusion

In conclusion, this study highlights the multifaceted nature of suicide attempts in prison and the impact of the COVID-19 pandemic. While preventive measures were essential for infection control, they may have increased psychological distress, and reduced medical resources likely exacerbated clinical needs. These findings underline the importance of balancing public health measures with continuous access to care in correctional settings. Protecting the health, dignity, and human rights of detained persons must remain a permanent commitment that extends beyond public health emergencies.

## Data Availability

The data associated with this study is not accessible to protect the confidentiality of the research subjects.

## Footnotes

1 Mental health problems include a range of symptoms such as anxiety, depression, personality disorders, drug/alcohol addiction, behavioral disorders, post-traumatic stress disorder, psychosis, sleep problems, adaptation problems, and other disorders. Other disorders include attention deficit and hyperactivity disorder, bipolar disorder, developmental disorders, low IQ, and somatoform disorder.

2 Psychotropic medications refer to a range of drugs that affect mental activity and behavior, including opiates, sleeping pills, sedatives, neuroleptics, antidepressants, mood stabilizers, psychostimulants, and antipsychotics.

3 Data were imputed through regression imputations (Zhang, 2016). Specifically, we developed a multivariable regression model for each missing item, using the missing item as an outcome and other study variables as predictors. After fitting the best model to the data, marginal effects were calculated to impute the value of the missing item for each detained persons, based their individual scores on the covariates included in the model.

4 Among the study participants who were not from Middle East–North Africa or Switzerland, 18.5% were from Europe and 12.2% were from another region.
